# Prediction of Cortical Bone Thickness Variations in the Tibial Diaphysis of Running Rats

**DOI:** 10.3390/life12020233

**Published:** 2022-02-03

**Authors:** Daniel George, Stéphane Pallu, Céline Bourzac, Rkia Wazzani, Rachele Allena, Yves Rémond, Hugues Portier

**Affiliations:** 1 ICUBE Laboratory, CNRS, University of Strasbourg, 2 Rue Boussingault, 67000 Strasbourg, France; remond@unistra.fr; 2 B3OA Laboratory, CNRS, INSERM, University of Paris, 10 Avenue de Verdun, 75010 Paris, France; stephane.pallu@univ-orleans.fr (S.P.); celine.bourzac@vet-alfort.fr (C.B.); rkia.wazzani@etud.u-picardie.fr (R.W.); hugues.portier@univ-orleans.fr (H.P.); 3 UFR Science and Technology, University of Orléans, 1 Rue de Chartres, 45100 Orléans, France; 4 DEPEC, Ecole Vétérinaire d’Alfort, 7 Avenue du Général de Gaulle, 94700 Maisons-Alfort, France; 5 APERE Laboratory, Université de Picardie Jules Verne, CEDEX, 80000 Amiens, France; 6 Laboratory Mathematics & Interactions J. A. Dieudonné, CNRS, University Côte d’Azur, 06108 Nice, France; rachele.allena@unice.fr

**Keywords:** bone density, cell activity, mechanical modeling, experimental validation, rat, treadmill running

## Abstract

A cell-mechanobiological model is used for the prediction of bone density variation in rat tibiae under medium and high mechanical loads. The proposed theoretical-numerical model has only four parameters that need to be identified experimentally. It was used on three groups of male Wistar rats under sedentary, moderate intermittent and continuous running scenarios over an eight week period. The theoretical numerical model was able to predict an increase in bone density under intermittent running (medium intensity mechanical load) and a decrease of bone density under continuous running (higher intensity mechanical load). The numerical predictions were well correlated with the experimental observations of cortical bone thickness variations, and the experimental results of cell activity enabled us to validate the numerical results predictions. The proposed model shows a good capacity to predict bone density variation through medium and high mechanical loads. The mechanobiological balance between osteoblast and osteoclast activity seems to be validated and a foreseen prediction of bone density is made available.

## 1. Introduction

Bone remodeling has been at the heart of many osteoarticular problems, such as ageing, osteoporosis, fracture, and bone disease, since long before the well-known Wolff’s Law (reprinted many times and recently updated [1]) was stated. Medical doctors and scientists have tried for many years to understand the basic principles of bone remodeling in order to accurately predict its evolution as a function of time based on mechanical and biological aspects, to evaluate the prognosis for adequate repair. As such, one of the first main parameters identified for bone remodeling was the externally applied mechanical load [1,2,3]. It is predominant in the way that bone structure must be fit to support the body weight and overall body functioning. Hence, changing the body weight or internal body functioning, impacts the bone density distribution. In order to predict bone remodeling as a function of an externally applied mechanical load, many models have been developed. Some of the known ones were developed by Beaupré, Ruimermann, or Pivonka [4,5,6], up to the well-known bone mechanostat developed by Frost [7,8]. However, most of these models are based on approaches where the bone microstructure evolution is dependent on a given “stimulus” being solely mechanically driven, usually under the form of strain energy. Yet, as a living material, bone will evolve not only from the mechanical point of view but also from the biological one, since organs and bones are dependent on living cell activities. For this reason, numerous works trying to link bone biology, bone mechanics, and the main biological factors of bone remodeling were published [9,10,11,12,13,14]. The difficulty in integrating cell activity with bone remodeling is mainly due to the fact that bone density is a macroscopic parameter (at the continuous scale of the bone), whereas cell biology occurs at a local scale, such as tens of microns. For this reason, some models have tried to bridge the different scales of bone remodeling (see, e.g., [15,16,17,18]). However, the limited knowledge of bone mechanobiological functioning remains a gap difficult to bridge to validate these models on human patients, particularly when accounting for patient dependency [19,20,21]. It is possible, at the macroscopic scale, to integrate more complex mechanical behavior in order to try accessing the local microstructure distribution that influences the bone density variation, either with or without an implant [22,23,24,25,26,27]. However, most of these models remain mechanically driven without accounting for the independent cell activity intensity that drives the bone remodeling.

In order to more precisely address this bone remodeling process from the simulation prediction point of view, one must account for both the mechanics (external forces applied to the structure) and the independent cell activity that are at play within the bone structure. A non-exhaustive list of the main influencing parameters for bone remodeling includes cell migration and differentiation, together with their interplay with their mechanical environment [28,29,30,31,32]. In addition, the mechanics should be coupled with the biology into a common bone “stimulus” leading the bone remodeling [33,34]. Although these effects are visible at the macroscopic scale of the bone, they are also dependent on local biophysics, such as cell functioning and differentiation, fluid flow, and biological transformations at the microscopic scale [35,36,37,38,39,40]. These aspects are difficult to integrate in a fully coupled model. Nevertheless, all these works support the conclusion that bone remodeling, both at macroscopic and microscopic scale, is dependent on a mechanical load imposed on the structure, either by gravity or by the muscles (even without gravity) that will drive a biological response through bone cell activity. These two steps are compulsory and independent and cannot be merged, as their fusion may lead to identical results with different couplings. Hence, any theoretical numerical model, even phenomenologically driven, that aims to represent bone remodeling should be based on this principle.

As such, assuming that the biological parameters (different cell activity) driving the bone remodeling can be identified experimentally, either under normal earth gravity or hypogravity [41,42,43,44,45] conditions. It could be possible to study/predict bone density variations (bone remodeling) as a function of time for different load conditions when providing an adequate coupling between the bone mechanical response and the cell reaction to drive the bone remodeling. In this work, we evaluate, from experimental data, a continuous theoretical numerical model based on George et al. [46] to study bone density variations under medium or high mechanical loads and compare these numerical results with experimental ones obtained from running rats. We show that the bone density either increases or decreases, depending on the running exercise intensity and that cell activity is directly related to this evolution. The first part or the paper is dedicated to the experiments and data extraction, and the second part is dedicated to the theoretical numerical simulations to validate the proposed numerical model. The results and discussion follow for the validation of the proposed predictive model.

## 2. Experiments

**Figure 1:**
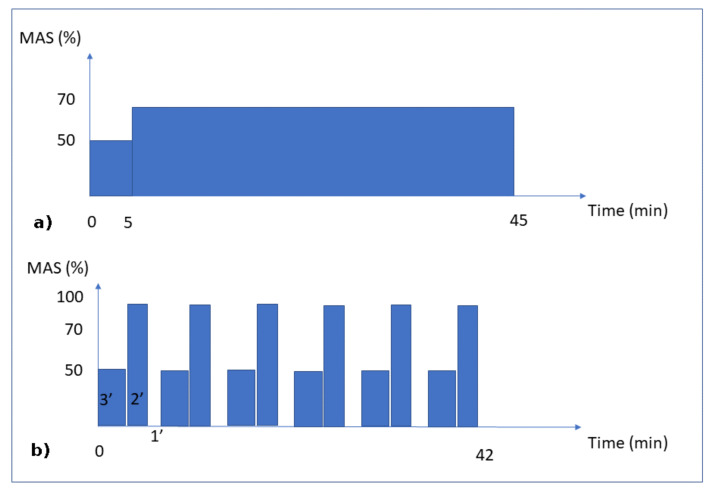
Training Protocols. Moderate Continuous running (**a**) and Interval running (**b**). MAS corresponds to the maximal aerobic speed.

Experiments were carried out on 3 groups of 7 initially 5-week-old male Wistar rats. The experimental protocol was approved by the Ethics Committee on Animal Research of Lariboisiere/Villemin (Paris, France) and from the French Ministry of Agriculture (Paris, France, reference number APAFIS # 9505). All rats were housed in controlled facilities (3 rats per standard cage, maintained on a 12-h light/dark cycle, at a constant temperature of 21 ± 2 °C). A commercial standard diet (Genestil, Royaucourt, France) and tap water were provided ad libitum to all animals. No significant variations were observed between the rats regarding their weight and sizes during housing. After one week of acclimatization, one week of treadmill running training was carried out (25 min per day to level off each rat) for the homogenization and practice scenario. The physical characteristics of the rats were then measured for each group (specifically, length and body weight). The rats were then submitted to one of the following scenarios:

1. Sedentary control group: the rats were 26.7 (±1.1) cm long and weighed 492.1 (±34.6) g. They were left to their everyday activity (eating and walking), without any specific running activity, for 8 weeks. At the end of this program, the mean cortical bone thickness of the tibial diaphysis was 957 (±110) µm. This value was normalized to 1 to facilitate comparisons between groups. The mechanical load (body weight) for the bone remodeling was assumed to be constant over time.
2. Continuous running group: the rats were 26.4 (±0.7) cm long and weighed 486.4 (±31.8) g. This group was subjected to 8 weeks of running for 45 min per day at an intensity of 70% of the maximal aerobic speed (MAS) (Figure 1a). At the end of the exercise program, the mean cortical bone thickness of the tibial diaphysis was 708 µm (±65 µm).
3. Intermittent running group: the rats were 26.5 (±0.6) cm long and weighed 475.1 (±30.3) g. This group was subjected to an interval-training running activity for 42 min per day for 8 weeks. This protocol consisted in 7 repetitions of blocks of 3 min at 50% of the MAS, followed by 2 min at 100% of the MAS and 1 min of passive rest (Figure 1b). At the end of the exercise program, the mean cortical bone thickness of the tibial diaphysis was 1024 (±112) µm.

Before and after the 8 week program, a progressive running test was used to determine the MAS for each rat [47]. Briefly, the test started with a 5 min warm-up (13 m/min) at a 10° inclination. Then, the speed was first increased to 17 m/min for 2 min and then by 4 m/min every 1min 30s. The test was conducted until fatigue occurred. Fatigue was defined as when the rats could no longer keep pace with the treadmill speed despite two consecutive stimulations with compressed air sprays. The test was then stopped, and the last fully sustained increment speed was defined as the MAS for the rat.

For the mechanical load conditions, the three groups were subjected to their own body weight. Since the body weight load conditions (whether running or not) lasted only a few seconds or less, while the bone remodeling process lasted days or weeks, we assumed that the intensity of the mechanical load remained at a constant body weight, disregarding the intermittent and inertial effects due to the impact of the legs on the ground while walking or running. The mechanical load (exercise intensity) on the bones was assumed to be directly dependent on the MAS intensity (linked to the aerobic capacity through oxygen uptake) for each group (Figure 1). Hence, we assumed that the continuous running group had a 70% level of exercise intensity, the intermittent running group (by intervals) had a maximum level of 100% exercise intensity, and the sedentary group had a 10% level of exercise intensity, corresponding to the average concentration of oxygen in the blood at rest classically described in the literature.

Since the final goal of this work was the numerical prediction of cortical bone thickness through the bone density variation as a function of physical exercise intensity, we wanted to be able to validate the numerical results with experimental data. We assumed that all rats were identical and that their normal bone growth was similar, as a function of time (ageing). Hence, there was no need to measure the variation in bone density at different times and compare them. We only needed to compare the sedentary control group (no exercise) with the two others (with exercise) to validate the variation in bone density as a function of exercise intensity.

Regarding the experimental part, a number of tests were carried out at the end of the eight week running scenarios. Post-mortem micro-computed tomography (µCT) was performed to determine the cortical porosity (a parameter for bone density) and variations in the bone microstructure as a function of time. Bone histology was performed in order to precisely evaluate the variation in cortical bone thickness after the 8 week-scenarios. Finally, the occupancy rate of osteocyte lacunae and tartrate-resistant acid phosphatase (TRAP) histochemistry were undertaken to quantify the capacity of bone degradation as a function of time and to correlate it with numerical cell kinetics.

### 2.1. Post-Mortem Micro-Computed Tomography (µCT) and Computation

The left proximal tibia of each rat was imaged using a high-resolution µCT (Skyscan 1172, Bruker, Belgium). For the microarchitectural parameters and bone mineral density, the µCT images were acquired at the following settings: source voltage 80 kV, source current 100 µA, matrix 1000 × 668, exposure time 175 ms, rotation step 0.3 degrees, frame averaging 10, filter aluminum 0.5 mm thick, and pixel size 9.81 µm. For the cortical porosity parameters, the µCT images were acquired at the following settings: source voltage 80 kV, source current 100 µA, matrix 2000 × 1336, exposure time 345 ms, rotation step 0.3 degrees, frame averaging 10, filter aluminum 0.5 mm thick, pixel size 5.00 µm. Images were reconstructed using NRecon software, 16 bits (v1.7.0.4, Skyscan). They were then segmented into binary images using simple global thresholding methods because of the low noise and good resolution of the data sets.

For the trabecular mircroarchitecture and the trabecular and cortical BMD, 200 slices were selected from the images immediately after the distal growth plate of the tibia. The bone mineral density was computed from their respective region of interest (ROI) for the trabecular and cortical bone using hydroxyapatite phantoms for calibration with CTAn software (v1.16.4.1, Skyscan) [48]. For the cortical porosity parameters, the µCT images were acquired at the following settings: source voltage 80 kV, source current 100 µA, matrix 2000 × 1336, exposure time 345 ms, rotation step 0.3 degrees, frame averaging 10, filter aluminum 0.5 mm thick, pixel size 5.00 µm [49]. These data were later transferred into the numerical model where the bone geometry was idealized (as each rat bone is different) and the bone density was averaged over all rats in each group and for the trabecular and cortical regions.

### 2.2. Bone Histology

The left tibiae were sectioned in 2 halves after fixation, and the proximal halves were dehydrated with a graded series of alcohols, embedded into polymethylmetacrylate (PMMA) resin, and transversally cut to a thickness of 200 µm with a diamond saw. The slides were then polished to a thickness of 100 µm using an alumina polishing solution and, finally, were stained by Stevenel’s blue (VWR Chemicals–PanReac Applichem) and van Gieson’s picrofuchsin (VWR Chemicals–RAL Diagnostics). The thickness of the cortical diaphysis was evaluated by a histomorphometric analysis. The images were obtained using a digital microscope (Keyence, VHX-2000) at 10× magnification. Using the dedicated Keyence toolbox, the cortical thickness was measured at eight different locations on three sections. The results obtained were averaged to obtain a mean cortical thickness per rat for the anterior, posterior, lateral, and medial locations and compared them with the numerical results obtained after the simulations.

### 2.3. Occupation Rate of Osteocyte Lacunae

To assess the occupation rate of osteocyte lacunae (ORL), pictures were obtained from the same slides using another microscope (Nikon, digital camera DXM 1200-F) at 40× magnification. Using Image J (National Institute of Health, 1987), the number of empty lacunae and lacunae containing an osteocyte (full lacunae) were determined in one randomly chosen 100 × 100 µm^2^ area, on the same 3 slides used for histomorphometry, for each location (anterior, posterior, lateral, and medial). The osteocyte lacunar density was expressed in osteocyte lacunae/mm2 and the ORL (%) was calculated as: the number of lacunae with an osteocyte / (the number of lacunae with an osteocyte + the number of empty osteocyte lacunae) × 100. The results (see Section 4.4) obtained from each location were added and then averaged from the three slides to obtain a mean ORL per rat.

### 2.4. TRAP Histochemistry

The distal tibial halves were decalcified with EDTA 177 g/L, pH 7.0–7.3 (Osteosoft, Merck KGaA, Darmstadt, Germany) for 4 weeks, embedded in paraffin, and cut to a thickness of 4–5 µm with a microtome. To assess bone resorption, slides were stained with tartrate-resistant acid phosphatases (TRAP, Acid Phosphatase kit, Sigma-Aldrich, Steinheim, Germany) and counterstained with hematoxylin after paraffin removal. For each rat, 4 transversal slides were randomly chosen and, for each of these slides, 3 pictures were randomly obtained, 1 for each of the following regions (trabecular bone, intermediate cortical-trabecular area, and cortical bone) at 500× magnification. A polygonal region of interest was manually drawn (TRAP activity is stained in red) and the surface of osteoclastic resorption (see Section 4.4) was automatically retrieved using VHX software (KEYENCE) [50]. The results obtained from each location and each slide were added to obtain a global surface of osteoclastic resorption for each rat.

### 2.5. Statistical Analysis

Statistical analyses were performed on the experimental results using a commercially available software (StatView, Version 5.0.; SAS Institute Inc). The descriptive statistics were reported as means ± standard deviations of the mean (SD) or medians (with 25th and 75th percentiles), according to the normality of the data distribution (Shapiro–Wilk test). The differences between the sedentary control, moderate continuous running, and intermittent running groups were then assessed using a Kruskal–Wallis test, then a Mann–Whitney U test, when applicable. The level of significance was set at *p* < 0.05. The obtained results were then compared to the numerical predictions to check, qualitatively, on the comparative tendencies and evolution trends for bone remodeling (see Section 4.4).

## 3. Theoretical Model

### 3.1. Theory

The model proposed here is based on the work by George et al. [46]. It is developed at a continuous scale to predict the equivalent bone density, either in the cortical or in the trabecular regions. Bone density variations evolve as a function of the identified cell activity developed within the bone structure under an applied mechanical load (here the body weight of the rats in the different running scenarios). The variation in mechanical loads leads to a variation in the biological responses of the bone cells within the bone, hence leading to a variation in bone density adjusting to the new mechanical load conditions through bone remodeling. We assume that cell activity is linearly dependent on the intensity of the elastic mechanical energy.

The combined cell activity leading the bone remodeling is the sum of the osteoblastic and osteoclastic cell activities within a given bone volume or representative volume element (RVE, assumed large enough for both activities to exist at the same time), as proposed by Delaisse et al. [51]. At homeostatic equilibrium, no bone remodeling occurs. This corresponds to everyday life activity. For medium mechanical energy (energy that is bigger than homeostasis but not overloading the structure), the sum is positive, leading to positive bone remodeling (through bone formation) and an increase in the equivalent bone density. This corresponds to an alternate running activity at a moderate intensity (moderate running two or three times a week for a healthy human). For higher mechanical energy (an overload of the bone structure is developed due to excessive exercise), the sum is negative, leading to a negative bone remodeling (or bone resorption). This corresponds to overtraining in humans. The bone is overloaded with intensive sport activity, and this leads to a degradation of the bone, as it is not able to biologically sustain such a load scenario over long periods of time.

The cell activity is assumed to be linearly dependent on the applied mechanical load up to a maximum level. For a given mechanical energy above the homeostasis equilibrium, both osteoblast and osteoclast activities representing a quantity of bone formed or resorbed during a given time frame are given in kg·m^−3^·s^−1^ (this unit will need to be translated into biological unit at a later stage with enlarged experimental data available) as they start increasing to reach a maximum value that is dependent on the cell density at a given position within the bone. Using this approach, a four parameter model can be proposed as follows:

(1) $$\begin{array}{ccccc} A_{ob}=k_{1}\cdot W+\rho_{bone}^{ini} & \mathrm{for}\;W<W_{1} & ; & A_{oc}=-k_{2}\cdot W+\rho_{bone}^{ini} & \mathrm{for}\;W<W_{3}\\ A_{ob}^{max}=A_{1}+\rho_{bone}^{ini} & \mathrm{for}\;W>W_{1} & ; & A_{oc}^{max}=-A_{2}+\rho_{bone}^{ini} & \mathrm{for}\;W>W_{3} \end{array}$$

where $A_{ob}$ and $A_{ob}^{max}$ are the osteoblast and maximum osteoblast activities, and $A_{oc}$ and $A_{oc}^{max}$ are the osteoclast and maximum osteoclast activities. The four parameters *k*_1_, *k*_2_, *A*_1_, and *A*_2_ are the cell activity parameters leading the kinetics of bone density change of formed/degraded bone. $\rho_{bone}^{ini}$ is the initial bone density in the homeostasis condition. *W* represents the elastic mechanical energy developed within the structure *W*_1_ and *W*_3_ represents the two energy thresholds for which: (i) under *W*_1_, the structure is not overloaded, (ii) above *W*_3_, the structure is mechanically overloaded, and (iii) in between *W*_1_ and *W*_3_, uncertain conditions are present where we define *W*_2_ to be the value for which the sum of osteoblastic and osteoclastic cells is zero, with an unstable equilibrium. Once the cell activities (four parameters) identified from the experiments and the theoretical model are defined, the bone density can be calculated, together with its corresponding Young’s modulus. It is a well-known fact that the determination of the Young’s modulus of bone from experimental data is well scattered through patients (animals, humans, type of bones, age, etc.). Therefore, we used a known relation, $E=E_{0}\cdot \rho_{bone}^{2}$ (Figure 1 and Figure 2 from [52]), that can be used to fit the experimental data available in the literature, where *E*_0_ is the cortical bone Young’s modulus. This can be adapted at a later stage with more precise experimental data.

The above proposed model is valid within any given small region of the bone structure, defined within a given RVE where the bone density variation is small (within the RVE) but needs to be validated over the entire bone geometry (where bone density can vary importantly). Although it is well known that bone remodeling or bone density variations originate from osteoblast and osteoclast cell activities, driven by the mechano-sensitive osteocytes, assuming a direct link between the mechanical energy developed and the corresponding cell activity over an entire bone is not adequate. If one assumes a constant bone density distribution through a medium, then this relationship is somewhat direct, as the bone density variation can also be assumed constant throughout. However, the current work focuses on a rat tibia. Hence, there is an initial bone density distribution through the medium that varies from near zero in the center part to near 100% in the outer cortical region. Assuming an exclusively energy-based approach would define a maximum cell activity in the cortical region and a minimum cell activity in the trabecular region, which is incorrect, as most cell activity is in the center part of the bone (bone marrow) where all cells and biology are located. The energy-based model does not account for these cells’ locations, as it is based only on a mechanical energy density that is bone-density-dependent. We observe that the bone density variation occurs mainly around the interface between the cortical and trabecular bone. If we assume that a minimum bone stiffness is required to develop the mechanical energy and build new bone, this means that although the cell activity is maximal at the central part of the bone, its bone construction effect remains limited (as bone density in the center part is small). It will increase where stiffness increases to reach a minimum level for the mechanical energy to lead the cell activation process. It is, therefore, necessary to introduce an extension of the initially proposed theoretical model to account for this effect.

From the previous argument, we may assume an optimum stiffness to cell activity ratio at the cortical-trabecular interface, and since it is not possible to modify the developed mechanical energy, as it is based on the material continuum mechanics, we may assume that this effect originates from the cell activities themselves. This argument is based on the idea that a variable intensity in cell activity exists as a function of bone region (due to a variation of bone stiffness or bone density). In our case, this variation is dependent on three zones as a function of the bone radius:

- In the trabecular bone (center part), cells are located in the bone marrow and are ready to be biologically activated. However, they are not very active, as they are far away from the cortical-trabecular interface and the mechanical support of the bone.
- Around the cortical-trabecular interface (on each side) is where the cell activity is at its maximum for the bone remodeling to occur.
- In the cortical bone, mainly osteocytes are present to sense the mechanical load, without osteoblasts and with a minority of osteoclasts; hence almost no bone remodeling occurs.

We will assume, on a first approximation, that cell activity is dependent on the cell density only with a predefined scenario that is dependent on bone density. To represent this through the cortical-trabecular interface, we hypothesized that the intensity of the cell activity could be defined with a non-linear evolution as:

(2) $${\left(\alpha -\rho_{bone}\right)}^{n}\cdot Cell\_activity$$

where ${\left(\alpha -\rho_{bone}\right)}^{n}$ transcripts the intensity variation in cell activity on each side of the cortical-trabecular interface as a function of the bone radius. Here, on the contrary to a cell activity that is directly dependent on the bone density, we obtain an opposite scenario where the cell activity is inversely proportional to the bone density through the cortical-trabecular interface. This is opposite to what is seen with the classical energy-based model, but cells are located in the trabecular region and not in the cortical one. Hence, the maximum cell activity cannot be located where the maximum bone density is. The value α is close to 1. The maximum value of this function is observed on the trabecular side (with α close to 1 and $\rho_{bone}$ close to zero). Oppositely, when $\rho_{bone}$ is close to 1 (cortical region), the cell activity is close to zero. The maximum cell activity (due to the combined effects of Equation (2) and bone density distribution) occurs in the region around the cortical-trabecular interface.

In addition, as cell biology differs between the intermittent and the moderate continuous running scenarios (with respects to the bone formation vs. bone degradation kinetics with two different mechanical energy levels), it may also be assumed that this cell activation curve (Equation (2)) through the interface is different between both scenarios. This was accounted for using the power n coefficient in Equation (2). Different n-values were identified for different energy levels, showing a strong influence of this parameter, which was validated experimentally (see the Section 4).

The proposed model can be used for a standard load scenario, together with increased loads being either biologically “healthy” or over-load cases. It could be extended further to under-load conditions, such as hypogravity, but accounting for different cell activity conditions. This will be achieved at a later stage.

### 3.2. Application

**Figure 2:**
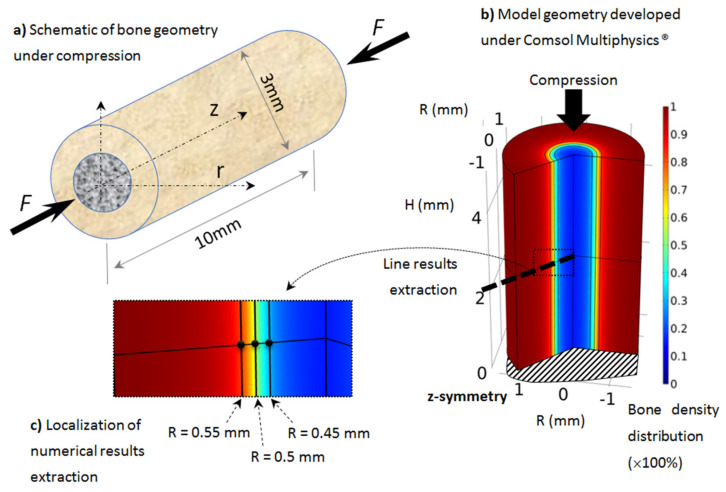
Developed axisymmetric model of bone, assumed to be cylindrical and under simple compression: (**a**) schematic of the model, (**b**) geometry, boundary conditionsand initial bone density distribution (going from cortical bone in red to trabecular bone in blue), (**c**) localization of numerical results extraction along a mid-height radius line and three points through the cortical-trabecular interface with same color bone density distribution.

As presented in Section 2, the proposed model was applied on rat tibiae (assumed similar for all rats but with different material parameters and load conditions). Although most long bones sustain a complex load scenario (tension, compression, and bending), as rat tibia is mainly vertical, we assume that most of the mechanical load is applied in the compression length of the bone, due to the body weight. The geometry was idealized from real bone with similar dimensions and assumed to be cylindrical and under simple compression (Figure 2).

The geometry was 10 mm long, with a 3 mm external diameter and a 1 mm cortical bone thickness (Figure 2a). We focused on the tibia diaphysis, where the cortical bone density and thickness were observed to vary in each scanned experimental sample as a function of the exercise intensity. The geometry was modeled using the Comsol Multiphysics^®^ software with a half-length 2D axisymmetric model (Figure 2b). The boundary conditions of the model (mechanical load and material parameters) were changed on the same model geometry and bone density evolution was then extracted.

**Figure 3:**
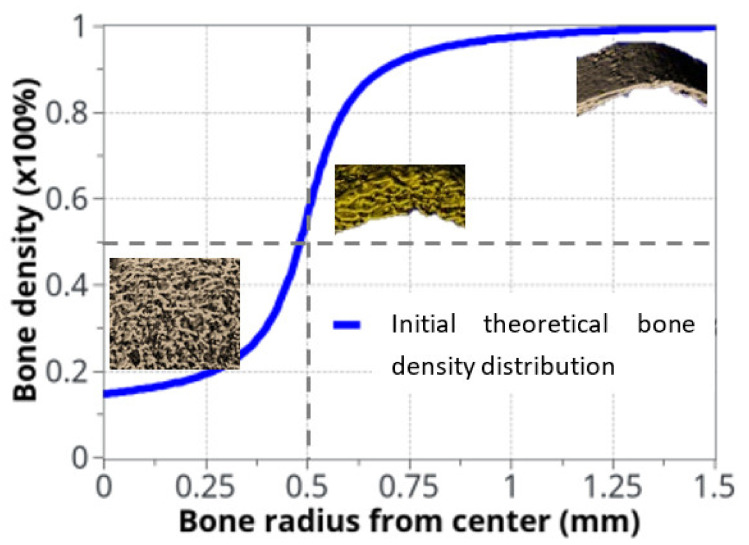
Idealized bone density distribution as a function of radius within the bone. The µCT measurements extracted from rats are also displayed. To keep continuity and transition between cortical and trabecular bone, the bone density variation as a function of radius was approximated with: $\rho_{bone}\left(R\right)=\left(\left(atan\left(12000*\left(R-0.0005\right)\right)\right)/3.4\right)+0.56$.

The variation in bone density was observed experimentally, mainly at the cortical-trabecular interface, slowly varying in time. Due to the sharp variation in bone density through this interface, an arbitrary nonlinear distribution, in the form of arctan function, was chosen in the continuous model for the bone density distribution as a function of radius (Figure 3). The form of the arctan function was chosen to be close to the experimentally observed bone density distribution. Other physical mechanical parameters were defined by the Young’s modulus of cortical bone: *E*_0_ = 20.3 GPa and Poisson ratio ν = 0.3 [53]. The applied mechanical load was provided by the rats’ body weight (350 g on average) and bone density was assumed with an idealized distribution (Figure 3) through the cortical-trabecular interface. It was also assumed to be a closed system with no external input.

.

When the elastic mechanical energy developed within the structure varies (as a function of the exercise intensity in the different running scenarios), bone density also changes (as a result of bone remodeling), mainly at the cortical-trabecular interface, due to the presence of osteoblast and osteoclast cells at this site. In the cortical region, osteocytes sense the applied mechanical load and transfer the mechano-sensing signal to the cortical-trabecular interface, where active cells are present. The numerical results will, therefore, be mainly extracted from this interface region (Figure 2c) at three different radii: R = 0.45 mm (trabecular zone), R = 0.5 mm (defined by the inflection point of the arctan function, or the mid-density of bone between the cortical and trabecular zones), and R = 0.55 mm (cortical region).

### 3.3. Parameter Identification

For the theoretical numerical model to run, it was necessary to identify two sets of parameters from experimental data: the different energy levels at which the cell activities change and the cell activities themselves (Equation (1)). The energy levels can be identified easily from the physics (rat body weight, femur geometry, etc.). They were identified in the following order:

- *W*_0_: Homeostasis, where no bone density variation occurs within the sedentary control group. It was evaluated directly from the rat body weight and bone geometry at the beginning of the experiments using the standard mechanics of elasticity.
- *W*_1_: Energy for the maximum mineral bone density increase, assumed to correspond to the bone density increase after 8 weeks of exercise in the intermittent running group. It was evaluated from the experimental data. This energy was extrapolated from the maximum cortical bone thickness observed experimentally in the intermittent running scenario.
- *W*_3_: The energy level corresponding to the maximum resorption rate (depending on cell availability), whatever the extra-increase in the mechanical energy. It was assumed to be the maximum degradation rate observed in the continuous running group after 8 weeks of running with the minimum cortical bone thickness.
- *W*_2_: The threshold energy level at which bone density will start to decrease if the energy level increases. It corresponds to the linear interpolation between W1 and W3, where osteoblast and osteoclast activity are considered to be equal.

Once the energy levels were identified, the cell activities needed to be identified using the experimental data. We started by considering the weight of the cortical bone [54], assuming an average value of 1 g·cm^−3^. As it was easy to extract experimentally the average volume of bone being formed or degraded in the two running scenarios. It was, therefore, easy to extract their corresponding weights. Hence, as the cell activity unit is defined in mg·mJ^−1^·mm^−3^·56 d^−1^ for *k_i_* and mg·mm^−3^·56 d^−1^ for *A_i_*, it is therefore straightforward to define what will be the corresponding cell activities for each energy level and time in the model from the mechanical energy (mJ) and exercise duration (56 days).

The results obtained for the different energy levels and model parameters are presented in the Section 4.

## 4. Results and Discussion

The body’s mechanical equilibrium depends on bone strength through bone mineral density and cortical bone thickness. The practice of a regular physical exercise is, therefore, healthy for the cardiovascular system as well as bone structure and stiffness reinforcement. However, overtraining may alter health conditions, as previously described [55,56,57,58]. The experimental results obtained in the current work confirm this observation. The averaged data obtained from the different running scenarios in rats provided evidence that cortical bone thickness decreases in the moderate continuous running group (bone resorption), while it increases in the intermittent running group (bone formation) (Table 1).

We observed an opposite relationship between the intensity of the developed averaged mechanical energy and the cortical bone thickness. However, the measure of the mineral bone density (Table 2) from the concentration of hydroxyapatite did not exactly follow this trend. Both the continuous and intermittent running groups showed a decrease in the hydroxyapatite concentration over time. The bone mineral density (BMD) in the two running groups decreased compared to the respective results in the sedentary group. Hence, no tendency conclusion can be drawn between the two exercise groups, since the BMD results are within the standard deviation.

A possible interpretation is that hydroxyapatite formation, a main determinant for bone strength, occurs at a different step of bone formation than bone density variation [12]. Both are linked but not at the same time. Hence, we may assume that a first step in bone remodeling could be a variation in the porosity within the bone structure, corresponding to a variation in bone density. Once this process is achieved, hydroxyapatite formation may develop to reinforce bone stiffness and bone mineral density. For the sake of simplicity, all the following data and particularly the numerical data will be interpreted as bone density changes as a function of porosity rather than hydroxyapatite concentration.

### 4.1. Numerical Model Parameter Identification

The two sets of parameters (Section 3.3) were identified (Table 3) from the experimental data (Table 1). It was assumed that the variation in cortical bone thickness (change in porosity) provided, over longer periods of time, the corresponding variation in bone density (change in stiffness) per unit volume. Hence, for a given body weight (load case) and bone geometry, it is possible to directly calculate the elastic mechanical energy developed within the bone and the model parameters.

**Figure 4:**
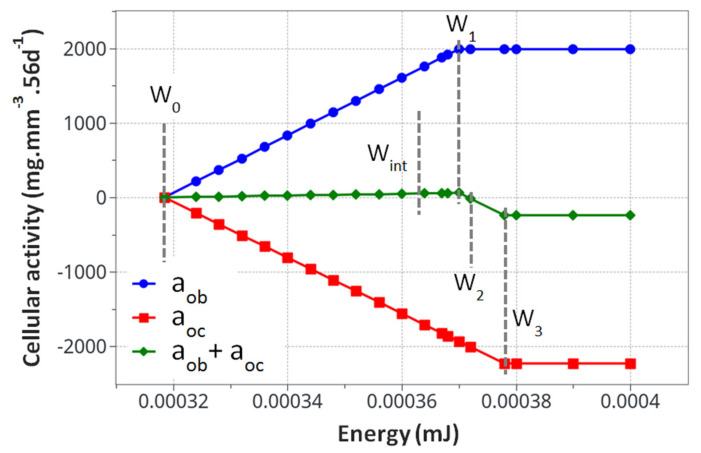
Calculated cell activity driving the bone density change. The evaluated parameters for the proposed mode *k*_1_, *k*_2_, *A*_1_and *A*_2_, are presented in Table 3.

The model kinematics (based on the Table 1 parameters) are summarized in Figure 4 with the knowledge that they correspond to the specific experimental case studied here without accounting for the initial osteoclast/osteoblast activation threshold.

From the cell activity curve (Figure 4) obtained from the identified model parameters, two observations can be made. First, the individual cell activity intensities (osteoblasts and osteoclasts) are large compared to their combined intensity driving the bone remodeling. However, since they are close to each other in terms of maximum intensity, their combined effects remain reasonably small. This can lead to several conclusions. Even with a potentially high cell activity, the bone remodeling phenomenon remains small, both in intensity and duration. For the bone density variation to become visible, either through exercise or medical effects, a long period of time will be necessary. Yet, as a subtle equilibrium is necessary for healthy bone functioning, any biological disequilibrium (variation in the two intensities between osteoblasts and osteoclasts) will lead to a quick disequilibrium in the bone remodeling kinetics. Hence, the variation of these parameters, either from ageing, diseases, or sport activity, may have a strong impact on the bone density.

The second observation is related to the form of the curve in Figure 4. It was assumed that the cell activities linearly depended on the mechanical energy (as classically assumed in the literature). This was, however, never validated experimentally, as the quantification of the intensity of bone cell activity as a function of mechanical load within an in vivo environment remains challenging. Hence, the linear dependency, even by validating the experimental observations (Figure 4), could be non-linear. This would, in essence, change the kinematics of bone remodeling as a function of time and should be investigated at a later stage.

### 4.2. Theoretical Model Results

For an optimum convergence of the numerical model, the parameters α and *n* required fine tuning for each of the different analysis cases. Table 4 shows the obtained results for the two energies, *W*_1_ and *W*_3_, of the intermittent and continuous running groups.

In the proposed relation ${\left(\alpha -\rho_{bone}\right)}^{n}$, the theoretical estimate of the α value should always be 1, since when bone density decreases towards 0, the cell activity is maximal (on the trabecular side of the interface); while when bone density is close to 1 (on the cortical side of the interface), minimal cell activity occurs. However, a numerical analysis showed that choosing 1 for all α did not lead to adequate results, or at least it did not provide results that correlated with the experiments. Hence, fine tuning of the different parameters was performed. This fine tuning needs to be investigated in future works. Since there is a strong sensibility of α, not only in its role in the cell activity intensity, but also in the long-term effect of the bone density evolution, a subtle variation in α can strongly affect the tendency to observe bone formation or bone resorption as a function of time at a given point of the bone structure. Hence, a careful adjustment is required in order to adequately fit with the experimental results. This is also due to the simplifying assumption of the continuous model.

The power coefficient, *n*, also plays an important role in the capacity of the bone to form or resorb. For example, using a high coefficient, n, for bone resorption (*W*_4_) leads to incorrect results. Hence, the curvilinear form of the cell activity as a function of bone density is highly important for bone remodeling kinetics. In other words, the intensity of the cell activity not only depends on the cell density at a given place and time but also on the mechanical environment (either through mechanical load or their structural basement) for the biological response. This is a well-known fact for cell activity response in the literature [59,60].

**Figure 5:**
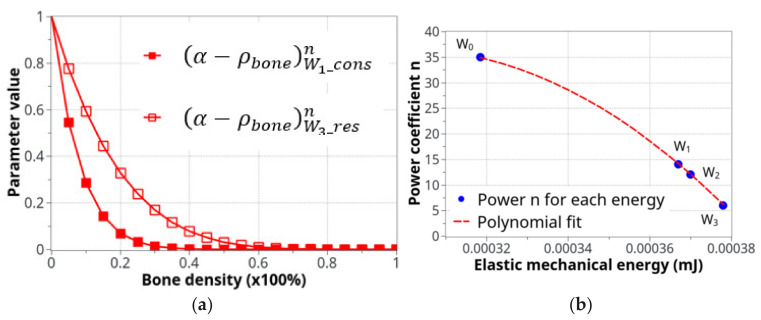
Parameters used for the definition of the cell activity intensity: (**a**) the coefficient of cell intensity as a function of the bone density for the construction and resorption phases at the two levels of energy, *W*_1_ and *W*_3_and (**b**) the power n coefficient of ${\left(\alpha -\rho_{bone}\right)}^{n}$ that defines the construction-resorption rates in the different running scenarios.

The two curves in Figure 5a show the evolution of Equation (2) as a function of bone density (high for low density and low for high density) and the specific difference in cell activity between the two energy levels of *W*_1_ and *W*_3_. In Figure 5b, the polynomial interpolation between the n parameters is presented for different levels of the developed elastic mechanical energy. Both Figure 5a,b present a simplified cell response as a function of bone density and mechanical energy. Of course, the real biological behavior is expected to be more complex, but this simplified approach provides adequate results compared to the experimental results and seems to be in agreement with the known cell behaviors involved in bone remodeling.

### 4.3. Bone Density Prediction

All parameters evaluated and integrated within the proposed mechano-regulatory model analyses were run for the different load cases (homeostasis, intermittent, and continuous running groups). In order to avoid overload of the Section 4, only the results for the two running scenarios (intermittent first and moderate continuous next) are presented here, for both the cell activity and bone density variations. Although the entire experimental time was 8 weeks, most of the bone remodeling (the biggest variation) occurred within the first 30 days, due to the model parameter identification (see lower down the interpretation of the cell activation delay). Only slow evolution, albeit non-zero, was observed for the rest. Hence, all results presented below will focus on the first 30 days of evolution.

#### 4.3.1. Intermittent Running Scenario

**Figure 6:**
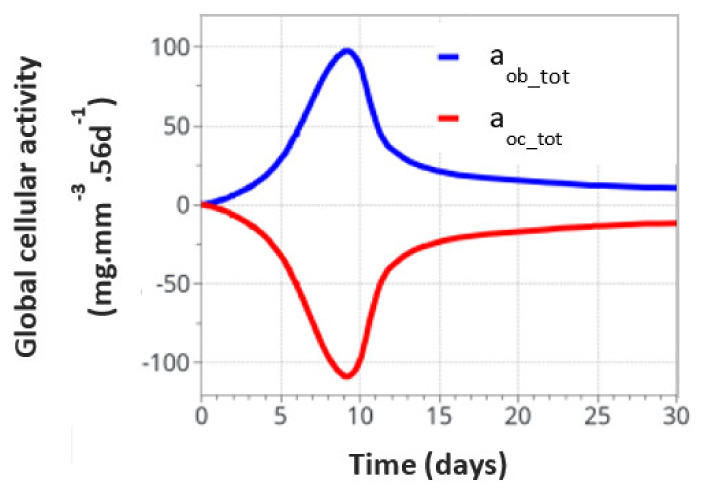
Global osteoblast and osteoclast activities over the entire model for the intermittent running scenario.

**Figure 7:**
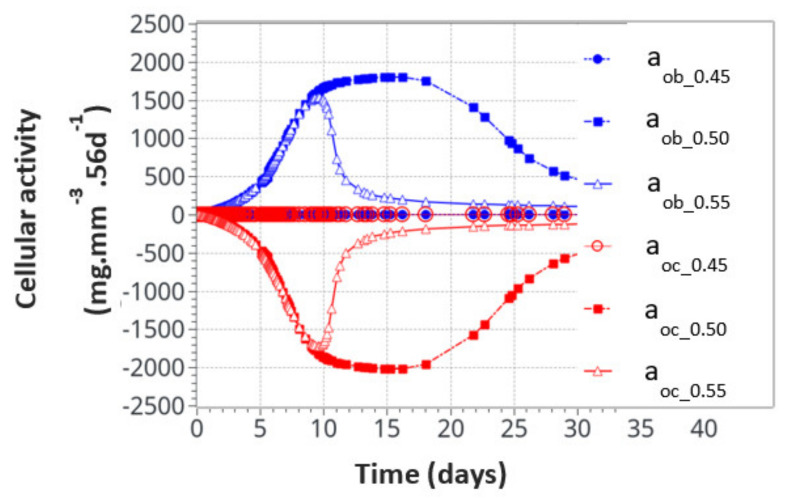
Osteoblast and osteoclast activities at the three locations through the cortical-trabecular interface. Measurements of the radii were 0.45 mm, 0.50 mmand 0.55 mm.

For the intermittent running scenario, both osteoblast and osteoclast activities increased at the same time, as a function of the applied mechanical load with a peak of activity around 10 days (Figure 6). Then, this activity slowly decreased towards equilibrium, as bone density had evolved to sustain the applied mechanical load. The global osteoclast activity seemed more important (around −110 mg·mm^−3^·56d^−1^) than the osteoblast formation (around +100 mg·mm^−3^·56d^−1^). The mistake to avoid here is to link this directly to the average bone density resorption. It only means that the maximum cell activity, which depends on both the geometrical location (hence the intensity of cell activity, as defined in Figure 5a) and the bone density, does not necessarily occur at the place where the bone density variation is at its kinetic maximum. Therefore, the average bone density variation may have different kinetics than the average cell activity. In Figure 7, we show the same running scenario and cell activities but at the three geometrical points located around the cortical-trabecular interface, as defined in Figure 2c. For the radius 0.45 mm, located in the trabecular zone, near-zero cell activity was found. However, maximum cell activity was observed at the mid-radius of 0.5 mm and decreased towards the cortical zone at 0.55 mm.

We also found that the cell activity time was much longer for the radius of 0.5 mm than for the radius of 0.55 mm. Thus, not only was the intensity of the cell activity higher at the mid-point location but also its time duration. With the highest cell activity located at the mid-point of the cortical-trabecular interface, it is then anticipated that bone density will evolve mostly around it.

**Figure 8:**
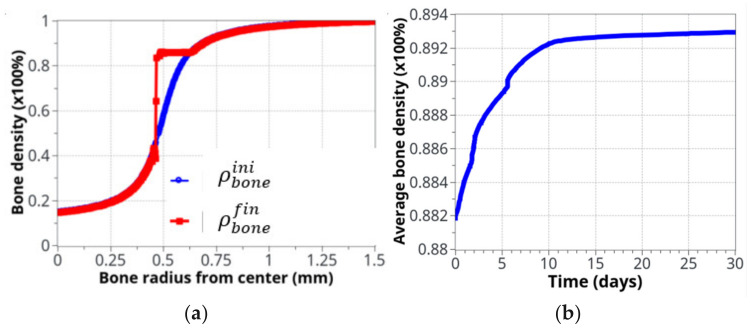
Bone density evolution: (**a**) initial (blue) and final (red), after 8 weeks running, distributions, as a function of radius; (**b**) The global evolution of bone density over the entire volume as a function of time.

Figure 8 shows the bone density evolution as a function of the bone radius (Figure 8a) and as a function of time (Figure 8b). As described above, the main evolution was located around the cortical-trabecular interface, as expected. Further away from this interface, the bone density was almost unchanged. This was due to the two following effects: (i) on the trabecular side, although cells are present and potentially active, the bone density is too weak (from the elastic mechanical energy point of view) to initiate this remodeling and (ii) on the other cortical side, although a high bone density may generate a high mechanical energy, the bone cells are almost inactive, due to their weak density. Hence, the highest combination is only located in the central part, where both effects are combined. The main difficulty here lies in the fact that bone cells are very sensitive to the mechanical load and bone density must be exactly located within the range that will activate the biological reaction (“activation zone”), otherwise bone remodeling does not initiate. A thin equilibrium is required to contribute to the bone formation.

If it is assumed that the initial cortical-trabecular interface is located at the inflexion point of the curve (radius = 0.5mm), the final state after bone remodeling places this interface around a radius of 0.45 mm. This can be achieved due to the identified model parameters (adjusted for this experimental case), the form of the cell activation curve (Figure 5), and the fact that the bone density far away from this interface leads to no variation (see above).

We can see the averaged (over the entire volume) bone density evolution as a function of time (Figure 8b). As expected from the cell activity (Figure 6 and Figure 7), the main variation occurred in the first 10 days, then, a slow evolution was observed. This 10 day period was too short compared to real experimental bone remodeling. This point is discussed further down as a function of cell activation time. Overall, the increase in bone density was about 1.5% compared to a theoretically calculated net change of 1.27%. The corresponding net weight change for a total volume of bone diaphysis of 125.6 mm^3^ is around 3 g.

**Figure 9:**
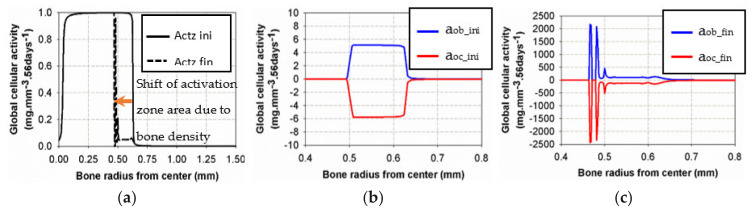
Evolution of osteoblast and osteoclast activities at the beginning (**b**) and the end (**c**) of the analysis for the intermittent running scenario compared to the initial and final “activation zone” (**a**).

Figure 9 shows the initial and final states of cell activities inside the bone, as a function of radius, together with the activation zone that corresponds to the area under which the cells can be active. At the beginning of the analysis, the “activation zone” (Figure 9a) covered the entire range of the trabecular and interface areas, down to zero in the cortical region (due to the assumed presence of only osteocytes in cortical bone). The initial osteoblast and osteoclast activities (Figure 9b) were of low intensity, centered over the cortical-trabecular interface. At the end of the analysis (Figure 9c), after bone reconstruction, intense osteoblast activity had shifted towards the trabecular zone, together with the “activation zone”, providing higher bone reconstruction on the trabecular side (low bone density) than on the cortical side (high bone density). We have particularly observed that the cell activities and bone reconstruction appear as localized events, meaning that they occurred within small zones (peaks), not continuously distributed over the entire bone structure. This means that not only the cell activity is localized but that changes in bone density are not necessarily constant throughout the height of the sample (see results below).

Although the final cell activities in the cortical side of the interface were higher at the end of the analyses than at the beginning, this did not generate more variations in bone density as the “activation zone” had moved towards the trabecular side of the interface with a low intensity in the initial area. This observation has a double meaning: (i) the intensity of the cell activity had a very low impact (Figure 5a) compared to the existing bone density that had increased at this time of analysis and (ii) this leads to a shift of the activation zone towards a lower bone density (trabecular side) where bone remodeling is still active.

**Figure 10:**
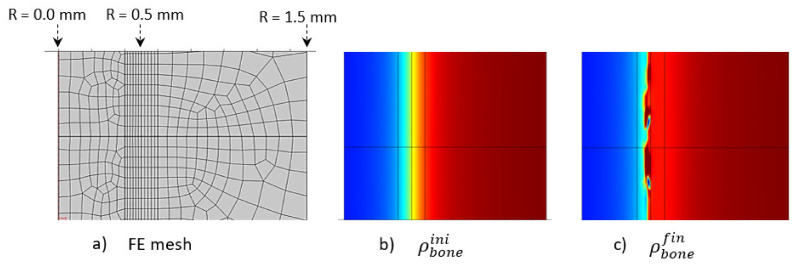
Initial (**b**) and final (**c**) (after 56 days running) bone density distributions with a corresponding finite element mesh (**a**) in the case of the intermittent running scenario.

The initially applied mechanical load was constant over the entire structure in the main longitudinal axis of the bone (diaphysis, vertical in Figure 10). A homogeneous mesh was defined around the entire cortical-trabecular interface (Figure 10a) to avoid any mesh dependency variations in the results. A convergence to a constant solution was achieved by running a mesh sensitivity analysis.

At the end of analysis after 56 days (8 weeks), an increase in global bone density was observed, with an average shift of the cortical-trabecular interface, as well as an increase in cortical bone thickness. This shift, however, was not constant over the entire bone length. Thus, it seems that, although the applied mechanical load was constant, the cell activity effect was not. Since the cell activity is geographically localized (Figure 9) and the cell sensitivity is very high, bone density may not evolve in a constant manner. A discrete local variation in stress impacts directly the bone density variation. Hence, a difference exists in the cortical bone thickness along the diaphysis length. An average variation in cortical bone thickness could be observed and showed good agreement with the experimental results (see below).

Another effect can be observed in the numerical results. Although the cortical bone thickness increased, it did not seem to grow over a maximum value (Figure 10c). There are several reasons for this. The first is the assumptions made for the proposed theoretical numerical model. For the bone remodeling to occur, it is needed for the cell activity to be in the range of activation energy, as defined in Figure 5a, and also for the adequate bone density in this area for the “activation zone” to be active at this particular point. Secondly, the maximum thickness limit is not only related to the modeling assumptions, as it is also correlated with the bone biology. Osteoprogenitor cells, which are present in the bone marrow, should be activated to differentiate in the osteoblast/osteocyte lineage and grow, but the cortical bone thickness (that is biologically made to support a given mechanical load scenario, based on earth’s gravity) cannot grow to a full cortical bone. Healthy biology prevents such a scenario to occur. Hence, although we developed a simplified model with coarse hypotheses, it appears to fulfill the basic biological requirements that need to be evaluated at a later stage.

#### 4.3.2. Continuous Running Scenario

**Figure 11:**
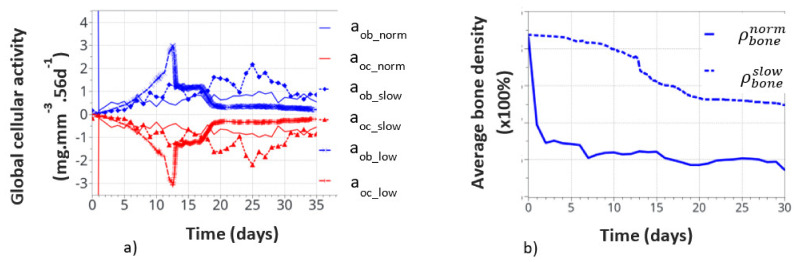
Global cell activity (**a**) and the corresponding bone density variation (**b**) for the continuous running scenario at different cell activity scenarios. The osteoblast (a_ob_norm_) and osteoclast (a_oc_norm_) activities correspond to the one defined in the above initial theoretical model.

Contrary to the intermittent running, where the cortical bone formed, continuous running degraded the cortical bone, due to the overload physiological context. Nevertheless, similar relationships develop within the structure between the applied mechanical forces and cellular activity, although opposite to what was observed in the intermittent running scenario. In addition, similar to the intermittent running scenario, cells are active only locally (from point to point of the structure, even with the continuous model scenario) and not in a homogeneous way. This effect is even more significant in the continuous running scenario. Hence, it is not possible to present a continuous cell activation curve through the thickness (as a function of radius) of the bone. We will then concentrate on the macroscopic results first. They are presented in Figure 11.

Figure 11a shows the variation of bone cell activity as a function of time, and Figure 11b shows the corresponding bone densities. We call the “normal” condition (norm), for which the model parameters were initially identified above. The bone cellular activity (a_x_norm_) responded instantaneously to the applied mechanical load. It increased quickly, as a function of time, up to about 10 to 15 days and then decreased slowly after bone remodeling occurred. For the continuous running scenario, this lead to a very quick bone density variation in a few days (see $\rho_{bone}^{norm}$ on Figure 11b).

This, of course, is not a realistic behavior, as bone density cannot change that quickly over such a short period of time. It is, therefore, concluded that immediate cell activation is probably not the best answer to represent bone remodeling. Hence, we assumed a time delay for the cell activity to develop due to the biological response to mechanical solicitations. This argument is supported by the fact that experimentally observed bone remodeling takes more time to be macroscopically visible. We delayed the bone cell response by adding a delay coefficient for this specific activity. To test this hypothesis, we defined two delay coefficients: (i) based on a time delay response, set to 1e^-5^ (“slow” variation), and (ii) based on a cell intensity response delay and time, set to 1e^-8^ (“low” variation). With these two coefficients, the corresponding cellular responses are showed all together in Figure 11a. The cellular responses were defined with the osteoblasts a_ob_slow_ and a_ob_low_, and osteoclasts with a_oc_slow_ and a_oc_low_. The time response and intensity of the cell activity were delayed in time, the and sensitivity of the biological response (peaks in the curves) also seemed lower. We also note that the time delay not only slows down the cellular response but it also slightly changes the evolution of the curve as a function of time. This is due to the fact that bone remodeling occurs locally as a function of the mechanical strain energy field, which is inhomogeneous within the structure. Hence, it impacts the cell activity scenario, although in a small amount, but it is still visible, which inevitably impacts the bone remodeling scenario over long periods of time.

The corresponding bone densities for the normal and slow cell activations are also presented in Figure 11b. Slower bone density kinetics were observed when bone cell activation was delayed, which seems to correspond better to real bone evolution. Bone density decreased by about 1% over 30 days, compared to over 1.5% for 7 days without a delay coefficient. These results support the idea of a time delay for the bone cell response. However, the final bone density distribution was similar for each of the normal, slow, and low resorption rates. Of course, this model is a simplified view of the real bone mechanobiology, and more work is required to argue on this effect. Nevertheless, the observed bone density kinematics seem similar when compared to the experiments. Assuming a time delay response of the cell activity also means that it is partially active for the intermittent running scenario but without knowing exactly its intensity. Hence, the resulting interpretation in the intermittent scenario remains partial for the time being.

**Figure 12:**
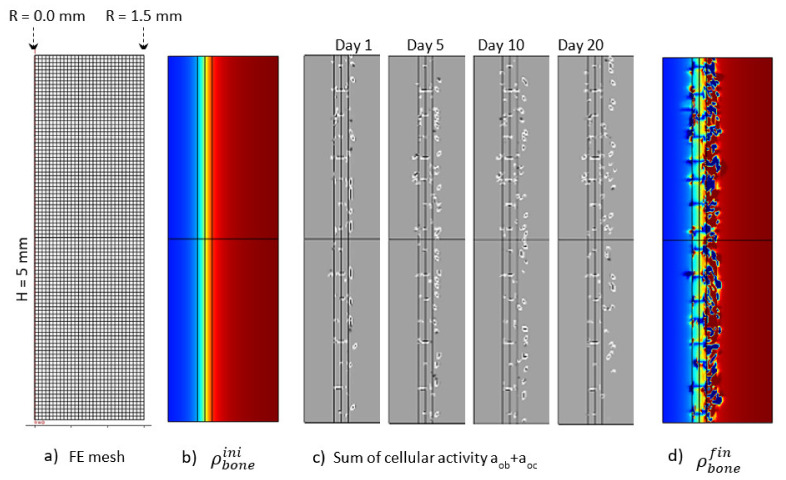
Initial (**b**) and final (**d**) (after 56 days running) bone density distributions, (**c**) cell activity a_ob_+a_oc_ at 1, 5, 10and 20 days of running, with the corresponding finite element mesh (**a**) in the case of the continuous running scenario.

The Figure 12 presents the macroscopic bone density distribution and cell activity, together with a finite element mesh. Since the cell activity was highly heterogeneous, a homogeneous mesh was defined through the entire geometry. Similarly, a mesh sensitivity analysis was carried out and the mesh size was decreased to a minimum level for which the bone resorption heterogeneity was not impacted by it. This led to a mesh element size around 100 μm. Osteoblast and osteoclast activity was highly heterogeneous, depending on the local mechanical stress at a specific point and time. This heterogeneity varied as a function of time (Figure 12c). The overall depth at which cell activity was observed increased towards the cortical side as a function of time. This was the main driving parameter of the bone resorption mechanism leading to the bone density distribution presented in Figure 12d. Of course, since it is a simplified continuous model, there is no representation of the bone microstructure evolution as a function of time. However, the bone density was not observed to degrade in a homogeneous manner. It can be argued that the heterogeneity of the bone density evolution is linked to the fact that the bone cell activity is directly related to the elastic strain energy at each point of the mesh and that a possible future development of the model would be to use some kind of averaging for the energy instead, as in [61,62], since the cells behave more like a network than a single entity. However, from the mesh sensitivity analysis, it occurred that even by decreasing the mesh size (smaller elements assuming a closer cell network), the size of the bone density resorption did not decrease anymore. Hence, this effect seems not to be at the origin (or it at least has a minimum effect) of the heterogeneous degradation.

Overall, over the entire length of the sample, bone resorption and a decrease in the cortical bone thickness were observed, according to experimental data. In addition, and similar to the intermittent running scenario, a ‘maximum’ resorption was observed, indicating that the assumed simplified model hypotheses seem to be validated by the result. A good overall correlation was observed between the numerical and experimental results. More work is, however, required to assess the influence of the different parameters.

**Figure 13:**
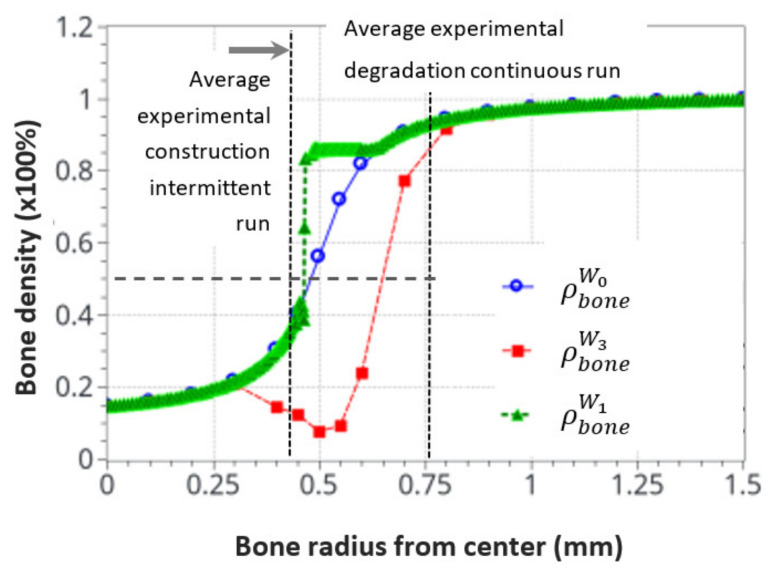
Initial and final average bone density distribution as a function of the radius through the bone. A comparison of the numerical predictions with the experimental results for the intermittent and continuous running scenarios. The dotted cross on the degradation curve corresponds to the median value of the considered cortical bone thickness after resorption.

Finally, Figure 13 shows the average bone density distribution as a function of the bone radius at the end (56 days) of the intermittent and continuous running scenarios compared to the initial distribution. The green average construction front located around the radius of r = 0.45 mm was compared with the dashed black line of the average experimental construction, located at r = 0.43 mm. Similarly, the red average resorption front located (at a mid-bone density, visualized by the bold dash line) around r = 0.65 mm was compared with the average experimental degradation front located at r = 0.76 mm.

Since the bone remodeling model proposed in this work is based on a continuous approach with simplified assumptions (idealized bone density distribution and simplified mechanobiological relationships), it could be expected that exact accurate comparisons between numerical and experimental results cannot be reached, as the bone microstructure and the influence of local biology were not accounted for. Nevertheless, a good correlation was observed between the numerical predictions and the experimental results. Both the formation and resorption effects were predicted as a function of the intensity of the mechanical energy applied and the cortical bone thickness variation. Even a complementary effect, highlighted by the heterogeneous bone density construction and resorption, was observed in the numerical results and was not initially anticipated in the continuous approach. This is consistent with a high influence of the bone microstructure distribution on the kinetics of bone remodeling rates.

It was also observed that both the new bone formation and resorption ranges were bounded by the modeling assumptions and available physics. These were the “activation zone”, the function of cell activity intensity, together with power coefficient, the bone density available at a given point and time, and the continuity (or discontinuity) of the bone density. In addition, only the bone cell activities, together with the elastic mechanical behavior, were accounted for. The intermittent running scenario showed a lower range of bone density variation (about 0.3–0.85), whereas the continuous running scenario showed a bone density resorption range (from 0.9 to about 0.1). This may be due to the simplified cell activation function defined in Figure 5.

Conclusions that can be drawn are numerous. The main driving parameters of bone remodeling could be obtained with a simplified mechanobiological approach, linking bone cell activity to elastic mechanical behavior. Of course, it does not mean these are the sole parameters, as bone mechanobiology is much more complex. However, the idea of a positive-negative interaction between the osteoblasts and osteoclasts seems validated for the maintenance of a bone remodeling equilibrium under a simplified mechano-regulatory behavior. The range of activity and intensity seems to be correlated with the experimental results. The change of the remodeling rate was highlighted as a function of cell activation time, and the range of bone density (in terms of depth and percentage between the trabecular and cortical compartments) discussed with the model assumptions. More work is required to fine tune these first predictive results by adding the influence of bone microstructure distribution and adjusting the model parameters and assumptions for better predictions.

### 4.4. Correlation between Cell Scale Experimental Results and Bone Scale Numerical Results

**Figure 14:**
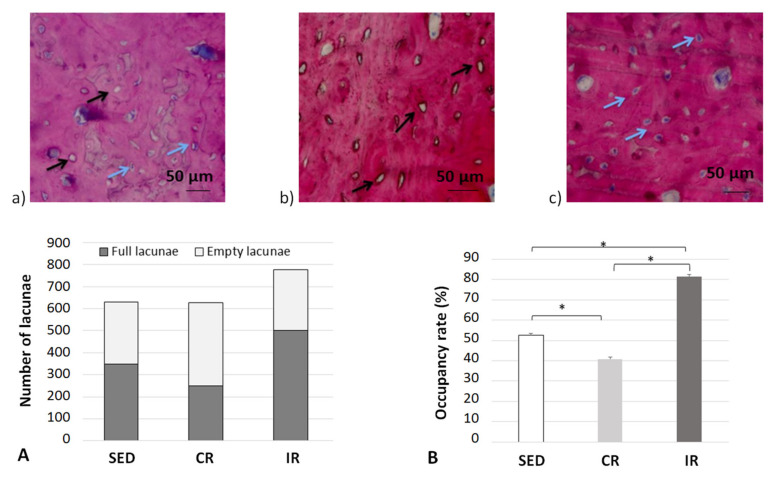
Occupation rate of osteocyte lacunae after 8 weeks of running in the cortical part of tibia. (**a**–**c**) show examples of cortical bone sections for the three groups with blue arrows for full osteocyte lacunae and black arrows for empty osteocyte lacunae. (**A**,**B**) generalize the results over all data sets. Sed: Sedentary control group, CR: continuous running group, IR: intermittent running group. Top panels (**a**) Sed; (**b**) CR, (**c**) IR. The blue arrows show a full lacuna (which contains an osteocyte), the black arrows show an empty lacuna. Bottom panel: (**A**) summarizes the number of full and empty lacunae in the three groupsand (**B**) includes the occupancy rate of the lacunae (%). Values are expressed as means of full and empty lacunae for (**A**) and as means +/− SD for B). *: *p* < 0.05.

It is now generally accepted in the literature that bone remodeling is the consequence of both osteoblast and osteoclast activities [51] through the signaling pathways developed between the mechano-sensing orchestrator osteocytes in the cortical bone matrix and transmitted to both the remodeling target cells. Hence, in order to evaluate the possible influence of this mechano-sensibility in our different running scenarios, the occupancy rate of osteocyte lacunae was measured experimentally at the end of the 8 week running scenario (Figure 14).

For the sedentary group, the quantity of empty osteocyte lacunae was significantly higher compared to both the intermittent and continuous running groups. In contrast, the full lacunae number was statistically higher in both running groups (*p* < 0.05). The lacunae occupancy rate significantly increased in the intermittent running group compared to the respective results in the two other groups and was 50% greater than the one observed in the continuous running group (*p* < 0.05). We may assume (see below) that, compared to the sedentary group, “overloading” the structure has led to a degradation by favoring a communication discrepancy between osteocytes and osteoblasts/osteoclasts, due to an increase (that needs to be defined) in osteoclast activity and, thus, bone resorption. On the contrary, the increased number of osteocyte-filled lacunae in the intermittent running group seem to favor communication with osteoblasts, hence favoring bone formation. One may wonder of the possible link existing between osteocytes and their connection types with current osteoblasts and osteoclasts.

**Figure 15:**
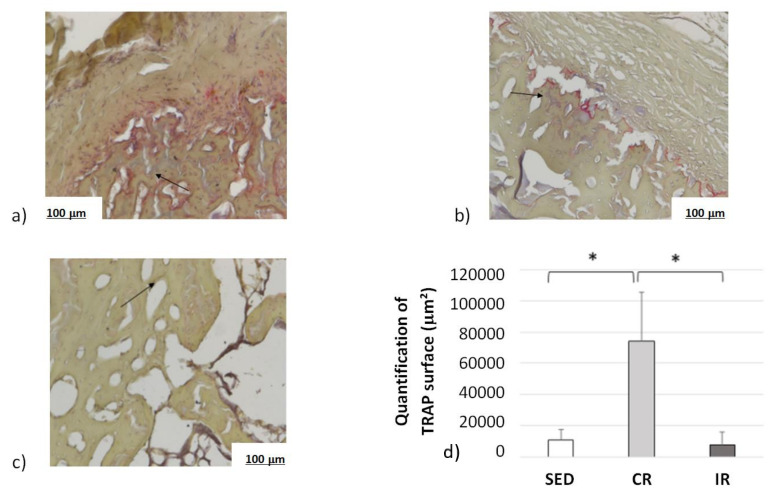
Osteoclast activity after 8 weeks of running in the cortical part of tibia. Sed: Sedentary control group, CR: continuous running group, IR: intermittent running group. Top panel (**a**) Sed; (**b**) CR, (**c**) IR. (**d**) The black arrows show the osteoclast TRAP activity (revealed in red). The results of the total resorption surface are given as means +/− SD (in µm^2^). *: *p* < 0.05.

Complementary information was found when measuring the osteoclast activity through TRAP analyses (Figure 15). Examples of surface of bone resorption are presented on Figure 15a–c for the sedentary, continuous, and intermittent running groups, respectively. The black arrows show in red the surface resorption area around empty spaces.

Averaged data over the entire surface of the cutting section for the three groups are presented in Figure 15d. The TRAP surface area (and thus the osteoclastic activity) in the continuous running group was much higher than in the two other groups. In addition, the areas of TRAP activity in the intermittent running group were lower than in the sedentary group. Here, again, the cell experimental results tend to confirm the corresponding activity for which a higher mechanical load intensity favored osteoclast activity, driving bone resorption, and a medium load intensity favored bone formation.

**Figure 16:**
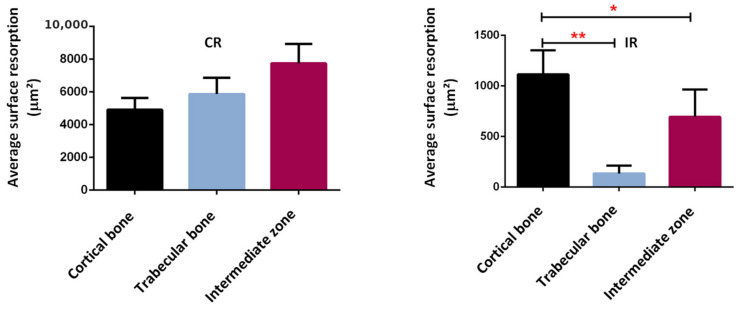
Average surface resorption in the different bone regions. *: *p* < 0.003, **: *p* < 0.0001.

Confirming these results, a bone resorption surface between 5000 µm^2^ and 8000 µm^2^ was observed in the continuous running group, while this surface represented between 200 µm^2^ and 1200 µm^2^ in the intermittent running group (Figure 16). Although some bone resorption occurred in the intermittent group, it was less pronounced than in the continuous running group and probably not high enough to compensate for the bone formation. In addition, when looking at the distribution of the areas of resorption activity, the maximum resorption in the continuous running group occurred in the intermediate zone (between the cortical and trabecular regions), followed by trabecular bone, and, lastly, the cortical bone. In the intermittent running group, the maximum resorption occurred in the cortical region (still much lower than in the continuous group), followed by intermediate zone, and, finally, the trabecular zone, which was close to zero.

These results tend to confirm two facts. Firstly, the assumption made for the theoretical model parameters, as presented in Figure 5, and the activation zone, as presented in Figure 9, seem relevant. The cell activity does not directly depend on the intensity of the developed elastic mechanical energy (bone density directed by the strain energy density (SED) function), but rather on a given distribution of cell density, itself depending on bone type and biology at a given place and time and whatever the bone stiffness is at this point and time. Secondly, although bone resorption was observed in both the continuous and intermittent running groups, it appeared that the corresponding bone formation intensity was also dependent on the local biology for each case. Hence, in the continuous running group, bone resorption occurred, as osteoclast activity was higher than osteoblast activity, while in the intermittent group, it was the opposite.

Overall, from the results and interpretations made from Figure 14, Figure 15 and Figure 16, we may assess that the comparisons between the experimental and numerical results in Figure 13 are similar. The optimal cell activity to stiffness ratio seems to be located within the cortical-trabecular interface, as defined by Figure 5 and Figure 9, and does not depend only on bone stiffness through strain energy density function, as classically presented in the literature. In addition, bone formation/resorption seems to occur as per a ratio of cell activity that is both dependent and independent of the elastic mechanical energy developed and leads to an intercellular communication driving the specific bone remodeling at a given space and time. In this study, we observed that cortical bone thickness increases at a medium load intensity and decreases for a higher load intensity (Figure 13).

## 5. Conclusions

We developed a theoretical numerical model for the prediction of cortical bone density variations in rat tibiae under intermittent and moderate mechanical loads. The model parameters were evaluated from experimental data, and the numerical results were well correlated to them. The experimental and numerical models showed that bone density variation does not directly depend on bone stiffness, as still commonly used in the literature for the numerical prediction of bone density. Rather, a clever bone mechanobiology is developed within the structure through specific cellular activity driving the bone density change. The proposed simplified model addressed in some simplified ways these intercellular communications in order to predict the corresponding macroscopic bone density evolution from the local cell activity variations. Although this model was developed at a continuous scale, it enables a good understanding of the mechanobiological phenomena and drives the way for better and more detailed predictive models. The next step is to integrate the influence of the local bone microstructure distribution in this analysis to improve patient-dependent predictions.

## Figures and Tables

**Table 1:** Experimental results obtained from the rat tests regarding the variations of the cortical tibial bone thickness measured in the first proximal half of the diaphysis, for the three groups.

	Sedentary Control	Continuous Running	Intermittent Running
Cortical tibial thickness (µm)	957 (±110) Normalized = 1	708 (±65) Normalized = 0.74	1024 (±112) Normalized = 1.07
Loading type	Constant load (body weight)	1 week for 25 min/day, 8 weeks for 45 min/day, Oxygen = 70% MAS	1 week for 25 min/day continuous, 8 weeks intermittent for 42 min/day, Oxygen = 50%, 100% MASand rest

**Table 2:** Experimental measure of the bone mineral density per the concentration of hydroxyapatite in the bone structure for the different experimental conditions (at the tibial proximal diaphysis).

	Sedentary Control	Continuous Running	Intermittent Running
BMD (g HA/cm^3^)	113.11 (± 4.12)	109.16 (± 4.31) *	106.13 (± 4.22) *

* *p* < 0,01 vs. Sedentary control; BMD: Bone mineral density; HA: hydroxyapatite.

**Table 3:** Parameters for the proposed theoretical model. Energies were determined from only the experimental data (rat weight, size of bone, test conditions, etc.). The *k_i_* and *A_i_* parameters were calculated from the experimental data after the completion of the running tests.

Parameters Determined from Experimental Data (×10^−4^ mJ)				Parameters Calculated (*k_i_* = mg·mJ^−1^·mm^−3^·56d^−1^; *A_i_* = mg·mm^−3^·56d^−1^)			
*W*_0_	*W*_1_	*W*_2_	*W*_3_	*k*_1_	*k*_2_	*A*_1_	*A*_2_
3.1847	3.7	3.72	3.78	386.64 × 10^5^	−375 × 10^5^	1992.36	−2232.38

**Table 4:** Tuned α and *n* parameters of the relation ${\left(\alpha -\rho_{bone}\right)}^{n}$ for the optimum reconstruction/degradation rate of bone density, as compared to the experimental results.

Osteoblast Activity Coefficient at *W*_1_	Osteoclast Activity Coefficient at *W*_1_	Osteoblast Activity Coefficient at *W*_3_	Osteoclast Activity Coefficient at *W*_3_	Cellular Activity Power at *W*_1_	Cellular Activity Power at *W*_3_
α = 1.0009	α = 1.0	α = 1.0007	α = 1.0	*n* = 13.6	*n* = 5

## Data Availability

Not applicable.
